# Rewarding Social Interaction in Rats Increases CaMKII in the Nucleus Accumbens

**DOI:** 10.3390/biomedicines9121886

**Published:** 2021-12-12

**Authors:** Inês M. Amaral, Laura Scheffauer, Angelika B. Langeder, Alex Hofer, Rana El Rawas

**Affiliations:** Division of Psychiatry I, Department of Psychiatry, Psychotherapy, Psychosomatics and Medical Psychology, Medical University Innsbruck, 6020 Innsbruck, Austria; ines.amaral@i-med.ac.at (I.M.A.); laura.scheffauer@student.i-med.ac.at (L.S.); angelika.langeder@student.i-med.ac.at (A.B.L.); a.hofer@i-med.ac.at (A.H.)

**Keywords:** CaMKII, nucleus accumbens, social interaction, cocaine, natural reward, drug reward, psychostimulants, conditioned place preference

## Abstract

Calcium/calmodulin-dependent protein kinase II (CaMKII) is known to be involved in the sensitized locomotor responses and drug-seeking behavior to psychostimulants. However, little is known about the contribution of CaMKII signaling in the nucleus accumbens (NAc) in natural rewards such as social interaction. The present experiments explored the implication of CaMKII signaling in drug versus natural reward. In the NAc of rats expressing cocaine or social interaction conditioned place preference (CPP), αCaMKII activation was induced in those expressing social interaction but not cocaine CPP. In order to investigate the role of NAc CaMKII in the expression of reward-related learning of drug versus non-drug stimuli, we inhibited CaMKII through an infusion of KN-93, a CaMKII inhibitor, directly into the NAc shell or core, before the CPP test in a concurrent paradigm in which social interaction was made available in the compartment alternative to the one associated with cocaine during conditioning. Whereas vehicle infusions led to equal preference to both stimuli, inhibition of CaMKII by a KN-93 infusion before the CPP test in the shell but not the core of the NAc shifted the rats’ preference toward the cocaine-associated compartment. Altogether, these results suggest that social interaction reward engages CaMKII in the NAc.

## 1. Introduction

The calcium/calmodulin (CaM)-dependent protein kinase family, which modulates a multitude of neuronal processes, is activated by Ca^2+^ influx through L-type and other ion-permeable channels [1]. Of the CaM-dependent kinases, calcium/calmodulin-dependent protein kinase II (CaMKII) is a highly expressed serine/threonine kinase whose α and β isoforms form dodecameric homo- and hetero-holoenzymes in vivo [2]. CaMKII is a key regulator of learning and memory [3], in particular the α-subunit-composed heteromer (αCaMKII) [4]. System analysis of addiction-related networks suggests that CaMKII is a central component that links several pathways implicated in the development of addictive behaviors [5]. Furthermore, it was proposed that the amplification of Ca^2+^ transmission and signaling by enhanced conductance through L-type Ca^2+^ channels might mediate cocaine-associated effects [1]. Indeed, evidence indicates that interfering with L-type Ca^2+^ channels and CaMKII signaling alters various psychostimulant-related behaviors [1,3,4,6,7,8,9,10,11,12,13,14]. In addition, the increase in extracellular dopamine (DA) in the nucleus accumbens (NAc) of cocaine-sensitized animals involves the induction of calcium- and CaMKII-dependent mechanisms [15]. Although the contribution of CaMKII signaling in the NAc to sensitized locomotor responses and drug-seeking behavior to psychostimulants has been studied extensively, relatively little is known about the contribution of CaMKII signaling in natural rewards, such as social interaction.

When rats were self-administering cocaine in an addicted-like way, operant social interaction inhibited cocaine self-administration and prevented the incubation of cocaine seeking [16]. Moreover, in a conditioned place preference paradigm (CPP), the presence of a social partner to interact with, in the compartment opposite to the one associated with cocaine, abolished cocaine preference during conditioning [17,18,19,20] after the conditioning to cocaine was already established [21], and during extinction of cocaine preference [17,22,23]. Thus, rewarding social interaction, when available in an alternative context, could shift the preference away from drugs of abuse. 

The mesolimbic DA system, in which the NAc is a central component, is recruited by both natural and drug reward behaviors [24,25,26,27,28]. However, within this system, the intracellular cascades activated by either natural reinforcers or psychostimulant drugs might be different [20,29]. Indeed, it has been shown that NAc cyclic adenosine monophosphate (cAMP)-dependent protein kinase (PKA) is implicated in the expression of psychostimulant reward-related learning [20,29,30]. Nevertheless, manipulations of the PKA pathway in the NAc did not alter the expression of non-drug reward learning [20,30,31,32]. 

In this study, we explored whether CaMKII in the NAc is involved in the expression of learning produced by either drug (cocaine) or natural reward (social interaction). Generally, chronic exposure to psychostimulants induces αCaMKII in the NAc [2,9,12]. In addition, activation of CaMKII in the NAc is associated with short-term abstinence from cocaine self-administration [33], with incubation of cocaine craving [34], and with cocaine-seeking behavior induced by drug priming in abstinent rats after extinction of self-administration [1]. However, the effects of a sub-chronic exposure to cocaine in CPP [35], a paradigm based on learning, on the activation of αCaMKII in the NAc was only reported for low cocaine doses, inducing a 16% increase in NAc-αCaMKII phosphorylation [36]. Therefore, we first compared αCaMKII activation levels in the NAc after cocaine or social interaction CPP.

Given the ubiquitous nature of CaMKII and its role in several basal neuronal and behavioral functions, the use of systemic CaMKII inhibitors is not considered a viable approach [2]. Therefore, it has been suggested that an indirect targeting of the mechanism of CaMKII induction, specifically to a subregion of the brain reward circuitry, could provide a better insight into the involvement of the CaMKII pathway in reward expression and, thus, avoid the complications associated with systemic CaMKII inhibition [2].

Here, we investigated the role of NAc CaMKII in the expression of reward-related learning of drug versus non-drug stimuli by inhibiting CaMKII through an infusion of KN-93 in the shell or core subdivisions of the NAc. The effects of CaMKII inhibition on the expression of concurrent CPP for cocaine and social interaction were assessed. In this study, interaction with a social partner was offered in the alternative compartment to the one associated with cocaine during conditioning. Cocaine and social interaction have similar rewarding values, leading to equal preference when they are assigned to opposite compartments of the CPP [17,18,19,20,37]. Therefore, a shift toward the cocaine or social interaction-associated compartment, as a result of CaMKII inhibition, could be indicative of the role of CaMKII in the NAc.

## 2. Materials and Methods

### 2.1. Animals

Male Sprague-Dawley rats (Janvier Labs, Le Genest-Saint-Isle, France), 6–7 weeks of age, weighing 150–250 g, were singly housed upon arrival to the animal facility. Water and pellet chow were freely available at all times. A continuous 12 h-light/dark cycle was applied, with all experimental procedures taking place during the light period.

Rats were habituated to handling for a minimum of 5 consecutive days (5 min handling/day). Behavioral experiments took place at the age of 8 weeks.

Experiments and surgeries were approved by the Austrian National Animal Experiment Ethics Committee (BMWF-66.011/0131-WF/V/3b/2016; BMWF-66.011/0040-WF/V/3b/2019).

### 2.2. Stereotaxic Surgery and Intra-NAc Core and Shell Inhibitor Infusion

Surgical procedures in rats were performed at the age of 7 weeks. Bilateral guide cannulae (Plastics One, 23G, Bilaney, Düsseldorf, Germany) were placed in the NAc core region (anteroposterior: ±1.6 mm, mediolateral: ±2.3 mm, dorsoventral: −7.2 mm; measurements given relative to bregma) or in the NAc shell region (anteroposterior: ±1.6 mm, mediolateral: ±2 mm, dorsoventral: −8.4 mm; measurements given relative to bregma). Guide cannulae were fixed to the animals’ skulls by two stainless steel screws and dental cement. Dummy cannulae were used to prevent blockage. Post operation, animals were provided with analgesia as well as a 5-day recovery period prior to behavioral testing.

KN-93 was prepared to a concentration of 12 µg/µL (dissolved in distilled water). On CPP test day, rats received a single infusion of KN-93 (6 µg/0.5 µL/side) [1] (Tocris Bioscience, Bristol, UK), a specific CaMKII inhibitor, or vehicle (distilled water). Infusions were performed for approximately 2 min and then the infusion cannulae were left in place for 3 min to prevent back-flow. Animals were kept in their home cages for 30 min before CPP testing was performed.

Correct cannula placement for animals that received infusions was ensured after the end of the behavioral experiments (Figure A2A).

### 2.3. Conditioned Place Preference Apparatus

An unplasticized polyvinyl chloride apparatus (64 cm width × 32 cm depth × 31 cm height) was used for behavioral experiments. The apparatus consisted of three compartments: a middle (neutral) compartment with white walls and white floor (10 × 30 × 30 cm each) connected to two conditioning compartments (25 × 30 × 30 cm each). Walls of the conditioning compartments were either vertically or horizontally striped in black and white and displayed stainless-steel floors with 168 holes of 0.5 cm diameter or 56 slits of 4.2 × 0.2 cm each. Conditioning compartments were connected to the neutral compartment via detachable doors.

Between sessions, the apparatus was cleaned using a 70% camphorated ethanol solution.

A video camera recorded the animals’ behavior and trajectory. The acquired footage was later analyzed for time spent in each compartment by the use of ANY-maze Video Tracking Software (Stoelting Europe, Dublin, Ireland). Social interaction conditioning sessions were also recorded to analyze the time paired rats spent in direct contact in the last conditioning session.

### 2.4. Conditioned Place Preference Protocol

- Acquisition of CPP

Prior to conditioning, rats were subjected to a pretest session (15 min), in which they were allowed to move freely within the apparatus and their natural preference for a compartment was assessed (day 1). Conditioning to the stimulus, social interaction or cocaine, was performed according to pretest results: the less preferred compartment in the pretest was paired with the stimulus during the conditioning sessions. Rats were conditioned four times to their assigned stimulus. Conditioning sessions (15 min) took place twice a day (min. 4 h between morning and afternoon sessions) for four consecutive days. Rats of the saline CPP group received saline intraperitoneal (i.p.) injections (1 mL/kg) in both compartments. I.p. injections of saline and cocaine (cocaine CPP group) (hydrochloride salt, Gatt-Koller, Absam, Austria, dissolved to a concentration of 15 mg/kg cocaine in 1 mL/kg of saline solution) were given immediately before placement of the rats in the cocaine-paired compartment. Animals conditioned to social interaction (social interaction CPP group) were placed in the social interaction-paired compartment with a weight-matched conspecific. Pairs were assigned in the first conditioning and remained the same for all conditioning sessions.

- CPP testing

On day six of the experiment, rats were placed in the neutral chamber and then allowed to move freely between all three compartments (15 min). After testing, the time spent in the stimulus-associated compartment was analyzed.

- Concurrent CPP protocol

In the concurrent CPP paradigm, rats were conditioned to one stimulus (e.g., cocaine) in one compartment of the apparatus and to the other stimulus (e.g., social interaction) in the other compartment of the apparatus. Again, a six-day protocol was executed with all behavioral sessions lasting 15 min: pretesting on day one of the experiment, four days of conditioning with two sessions/day (e.g., social interaction in the morning and cocaine in the afternoon; min. 4 h between sessions), and CPP testing performed on day six.

As an additional measure, the time paired rats spent in direct physical contact during the last social interaction conditioning session was quantified by a researcher blind to the experimental groups. 

### 2.5. Western Blotting

Animals were euthanized by CO_2_ inhalation overdose 20 min after CPP testing. Brains were removed and immediately frozen. Brain sections were obtained using a cryostat (coronal sections of 150 µm) and NAc tissue was collected with a sample corer (Fine science tools, 11G–17G, Heidelberg, Germany). Samples were kept at −80 °C until further use.

Total protein was extracted from the collected NAc tissue. Briefly, brain tissue was homogenated in RIPA lysis buffer (Thermo Fisher, Scientific, Vienna, Austria) supplemented with protease and phosphatase inhibitors (100X) (Thermo Fisher, Scientific, Vienna Austria). This step was followed by mixing the homogenated tissue in an orbital mixer (30 min, 4 °C), and a centrifugation at 13,000× *g* (15 min, 4 °C). The supernatant containing the total protein extract was collected, and the protein concentration was quantified using a Bradford protein assay.

In preparation for western blotting, Roti-load buffer (Lactan, Graz, Austria) was added to protein extracts and samples (10–20 µg) were loaded onto a 10% acrylamide gel. After SDS-polyacrylamide gel electrophoresis, samples were transferred to a PVDF membrane and blocked for 1 h at room temperature (RT) in 5% non-fat dry milk (NFDM) in 0.1% Tween 20 in Tris-buffered saline (TBS-T) solution for non-phosphorylated proteins or 5% bovine serum albumin (BSA) in TBS-T for phosphorylated proteins. After blocking, incubation with primary antibodies took place (overnight, 4 °C) as follows: CaMKII (1:1000, Cell Signaling Technology Europe (CST) #4436, rabbit monoclonal), p-CamKII (T286) (1:1000, CST #12716, rabbit monoclonal), and β-III Tubulin (1:50,000, Novus Biologicals #NB100-1612, chicken polyclonal) as a loading control. Primary antibodies were diluted in 5% NFDM/BSA in TBS-T.

The next day, membranes were washed in 1% NFDM/BSA in TBS-T (3 × 10 min at RT) and incubated with either anti-chicken or anti-rabbit IgG horseradish peroxidase-conjugated secondary antibodies (1:20,000 in 1% NFDM/BSA, 1 h at RT). After washing (3 × 10 min), membranes were incubated with an enhanced chemiluminescence substrate (Bio-Rad, Vienna, Austria) and developed using a Chemidoc Imaging System (Bio-Rad).

The bands’ intensity was quantified using the Image Lab Software (Bio-Rad). Western blot results presented are shown as relative intensity of the ratio between the phosphorylated protein [p-CaMKII] and the loading control (β-III-Tubulin).

### 2.6. Statistical Analyses

GraphPad PRISM Software (GraphPad Software; San Diego, CA, USA) was used for all analyses, with data being shown as mean ± standard error of the mean (SEM). *p* values were considered statistically significant if they were < 0.05.

The significance between two experimental groups was tested using a two-tailed unpaired Student’s *t*-test. To test the statistical difference between three experimental groups, one-way analysis of variance (ANOVA) was used, followed by a Newman–Keuls multiple comparisons post-hoc test. Cohen’s *d* was calculated to evaluate effect sizes.

## 3. Results

### 3.1. Social Interaction CPP Increased CaMKII Activity in the Nucleus Accumbens

To study the effects of drug and natural reward on the activation of CaMKII in the NAc, a cocaine or social interaction CPP was performed.

**Figure 1:**
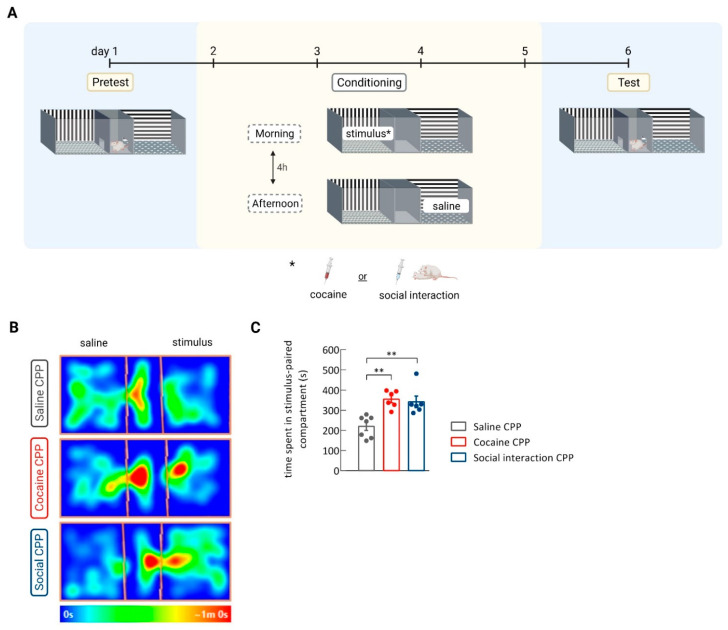
Conditioned place preference to cocaine or social interaction. (**A**) Timeline. (**B**) Representative heatmaps of the time spent in each compartment during the CPP test of rats receiving saline injections in both compartments of the CPP (saline CPP), and rats conditioned to cocaine (cocaine CPP) or social interaction (social CPP). (**C**) Time rats spent in the saline-, cocaine- and social interaction-associated compartments during the CPP test. ** *p* < 0.01, one-way ANOVA followed by Newman–Keuls multiple comparisons test; *n* = 6–7. Results are presented as mean ± SEM.

After the pretest, rats were trained to express preference for either cocaine (cocaine CPP group) or social interaction (social interaction CPP group) by an i.p. injection of cocaine (15 mg/kg) or being allowed to interact with a conspecific of similar weight, respectively. An additional group was defined as control, in which rats received saline i.p. injections in both compartments of the apparatus. After four sessions of conditioning for each stimulus, the expression of CPP was determined (Figure 1A). Rats conditioned to cocaine and social interaction spent more time in the stimulus-paired compartment as compared to the saline control group. An equivalent rewarding effect by cocaine and social interaction was observed, as there was no difference between cocaine CPP and social interaction CPP groups in the time spent in the stimulus-associated compartment during the CPP test (one-way ANOVA, treatment effect, F_(2,16)_ = 11.55; *p* = 0.0008; *n* = 6–7. saline CPP vs. cocaine CPP: ** *p* < 0.01, Cohen´s *d* = −2.81; saline CPP vs. social interaction CPP: ** *p* < 0.01, Cohen´s *d* = –1.99; cocaine CPP vs. social interaction CPP: *p* = non-significant (ns)) (Figure 1B,C).

**Figure 2:**
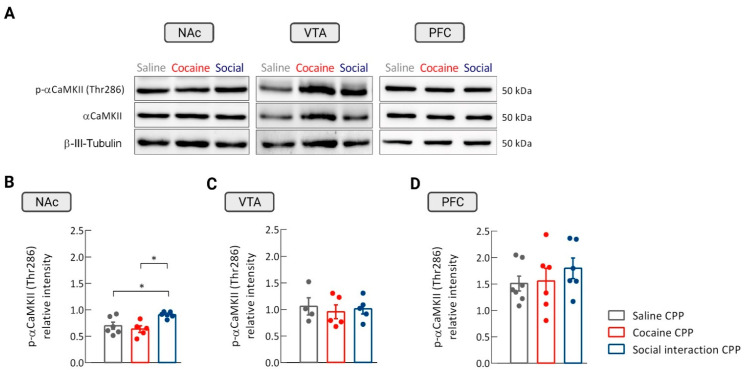
αCaMKII phosphorylation levels in the nucleus accumbens (NAc), ventral tegmental area (VTA), and prefrontal cortex (PFC) after cocaine and social interaction CPP. (**A**) Representative western blot images of phosphorylated α-CaMKII (p-αCaMKII) (Thr286), αCaMKII, and β-III-Tubulin levels (used as protein loading control) in the NAc, VTA, and PFC of rats from the saline, cocaine, and social interaction CPP groups. (**B**) Relative intensity of NAc αCaMKII phosphorylation at Thr286 in rats that expressed saline, cocaine, and social interaction CPP. * *p* < 0.05, one-way ANOVA followed by Newman–Keuls multiple comparisons test; *n* = 5–6. (**C**) Relative intensity of the bands that express p-αCaMKII levels in the VTA of rats from the cocaine and social interaction CPP groups, as well as the control group—saline CPP; *p* = non-significant (ns), *n* = 4–5. (**D**) Relative intensity of the bands that express p-αCaMKII levels in the PFC of rats that expressed cocaine and social interaction CPP, and the control group—saline CPP; *p* = ns; *n* = 6–7. Results are presented as mean ± SEM.

The role of CaMKII in drug and natural reward was subsequently investigated by assessing the effects of cocaine and social interaction reward on the expression of CaMKII in the NAc. For this, we performed a western blot analysis of total protein extracts from NAc tissue obtained from rats that expressed cocaine or social interaction CPP. An increase in the relative intensity of phosphorylated αCaMKII (p-αCaMKII) at Thr286 was observed in the group of rats that expressed social interaction CPP as compared to the saline control group. No differences were detected in p-αCaMKII levels between the cocaine CPP and the control group (one-way ANOVA, treatment effect, F_(2,13)_ = 5.75; *n* = 5–6. *p* = 0.0163; saline CPP vs. cocaine CPP: *p* = ns; saline CPP vs. social interaction CPP: * *p* < 0.05, Cohen´s *d* = −1.57; cocaine CPP vs. social interaction CPP: * *p* < 0.05, Cohen´s *d* = –2.41 (Figure 2A,B)). 

**Figure A1:**
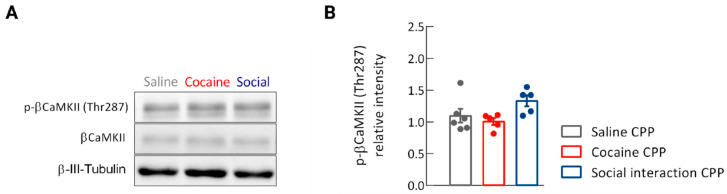
βCaMKII phosphorylation levels in the nucleus accumbens (NAc) after cocaine and social interaction CPP. (**A**) Representative western blot images of phosphorylated β-CaMKII (p-βCaMKII) (Thr287), βCaMKII, and β-III-Tubulin levels (used as protein loading control) in the NAc of rats from the saline, cocaine, and social interaction CPP groups. (**B**) Relative intensity of NAc βCaMKII phosphorylation at Thr287 in rats that expressed saline, cocaine, and social interaction CPP. *p* = ns; *n* = 5–6. Results are presented as mean ± SEM.

The phosphorylation levels of β-CaMKII in the NAc were also investigated. The relative intensity of phosphorylated βCaMKII isoform (Thr287) in the NAc did not differ between the groups of rats that expressed cocaine CPP, social interaction CPP and the control group (one-way ANOVA, treatment effect, F_(2,13)_ = 3.266; *n* = 5–6; *p* = 0.07 (Figure A1A). 

Additionally, the role of CaMKII in the expression of drug and natural reward in other reward-related brain regions, such as the ventral tegmental area (VTA) and the prefrontal cortex (PFC) was assessed. For that, a western blot analysis for p-αCaMKII (Thr286) levels in the VTA and PFC of the cocaine and social CPP groups was performed. No difference was found in the expression level of our kinase of interest in these groups when compared with the saline CPP group in both regions (one-way ANOVA; VTA: treatment effect, F_(2,11)_ = 0.1588 ; *p* = 0.86, *n*= 4–5; PFC: treatment effect, F_(2,16)_ = 0.6472; *p* = 0.54, *n* = 6–7 Figure 2A,C,D).

### 3.2. CaMKII Inhibition in the Nucleus Accumbens Shell Shifted Preference toward Cocaine

To clarify the role of NAc CaMKII in the expression of drug and natural reward, we explored the effects of CaMKII inhibition on the expression of cocaine and social interaction CPP. As the two subregions of the NAc—core and shell—are recognized to be differentially involved in reward-related learning [38,39,40,41], we looked at the effects of CaMKII inhibition in each of these two regions on the expression of drug *versus* non-drug reward learning.

**Figure 3:**
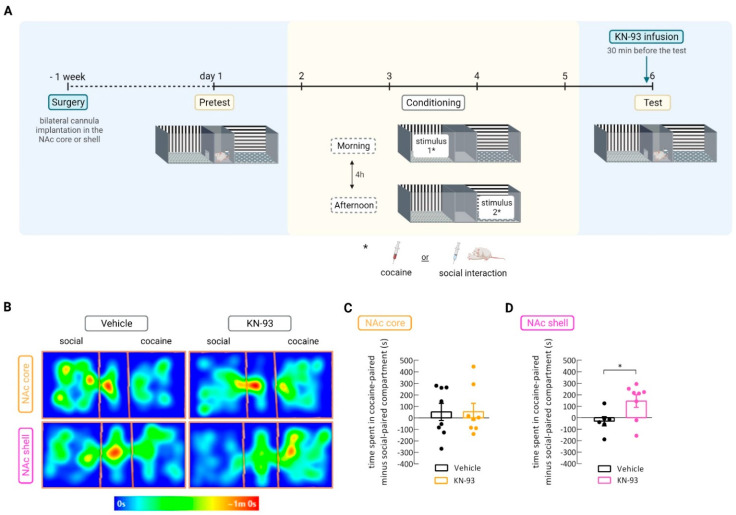
CaMKII inhibition in the NAc shell shifted the preference toward the cocaine-associated compartment. (**A**) Timeline. (**B**) Representative heatmaps of the time spent in each compartment during the CPP test of rats infused with vehicle or KN-93 in either the shell or core NAc subregions. “Social” and “cocaine” refer to the compartment side of the apparatus where the animals were conditioned with the stimulus before being tested for CPP. (**C**) Difference between the time rats spent in the cocaine- and in the social-associated compartments during the CPP test for rats infused with either vehicle or KN-93 in the NAc core; *p* = ns; *n* = 8. (**D**) Difference between the time rats spent in the cocaine- and in the social-associated compartments during the CPP test for rats that received an infusion of either vehicle or KN-93 in the NAc shell; * *p* < 0.05, unpaired *t*-test; *n* = 6–10. Results are presented as mean ± SEM.

In this experiment, social interaction was made available in the opposite compartment to the one in which rats were conditioned to cocaine. As established before, these two stimuli have a similar reward value (Figure 1C, vehicle groups in Figure 3C,D). Therefore, manipulation of CaMKII activation could shift the rats’ preference toward a specific stimulus-associated compartment in a concurrent CPP paradigm. 

Inhibition of CaMKII through a single infusion of KN-93 in the NAc shell (two-tailed unpaired *t*-test, * *p* = 0.03, t = 2.407, df = 12; Cohen’s *d* = –1.34; *n* = 6–8 (Figure 3D)) but not in the core (two-tailed unpaired *t*-test, *p* = 0.99 (ns), t = 0.01, df = 14, *n* = 8 (Figure 3C)), before the CPP test shifted the preference toward the cocaine-associated compartment, as shown by the increase in the time spent in this compartment during the test (Figure 3).

**Figure A2:**
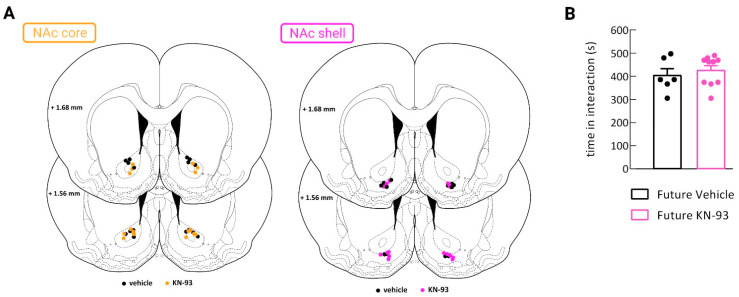
(**A**) Representative image of the infusion sites in the NAc core or shell for rats infused with either vehicle or the CaMKII inhibitor KN-93. (**B**) Time of direct contact in paired rats that received an infusion in the NAc shell during the last social interaction conditioning session. “Future Vehicle” and “Future KN-93” groups refer to the rats that received an infusion of Vehicle or KN-93, respectively, in the NAc shell, before being tested for CPP, *p* = ns; *n* = 6–10. Results are presented as mean ± SEM.

In order to investigate whether the observed increase in the time spent in the cocaine-associated compartment during the CPP test of rats infused with KN-93 in the NAc shell was due to a decrease in their initial interest for social interaction, we quantified the time paired rats spent in direct physical contact [42] during the last social interaction conditioning session, performed 24 h before CPP testing [20,37]. As no differences were detected in time spent in direct contact between the vehicle and KN-93-infused group of rats, the shift to cocaine preference observed in the KN-93 group was not the result of a decrease in social interaction (two-tailed unpaired *t*-test, *p* = 0.53 (ns), t = 0.6376, df = 14; *n* = 6–10 (Figure A2B)).

## 4. Discussion

The main finding of this study is that αCaMKII activation was induced in the NAc of rats expressing social interaction but not cocaine reward. Moreover, we found that inhibition of CaMKII by a KN-93 infusion before the CPP test in the shell but not in the core of the NAc shifted the rats’ preference toward the cocaine-associated compartment when social interaction was made available as an alternative to cocaine during conditioning. These results suggest that CaMKII in the shell subdivision of the NAc is implicated in the expression of social interaction reward.

αCaMKII expression in the NAc shell was reported to be increased after chronic use of cocaine [2,9] and amphetamine [12], and after reinstatement of cocaine seeking [1]. In addition, αCaMKII activated by chronic cocaine experiences was positively correlated with the motivation for the drug [9]. Our results show that sub-chronic exposure to cocaine at a dose of 15 mg/kg does not increase αCaMKII in the whole region of the NAc. It appears that a chronic rather than a sub-chronic cocaine exposure is necessary to induce αCaMKII activation specifically in the shell subregion of the NAc. However, Kong and colleagues could observe a slight activation of αCaMKII in the NAc after CPP with a low cocaine dose (2.5 mg/kg) [36]. Next to different cocaine doses used, the discrepancy between these observations might be due to the length of the CPP protocol performed in the two studies (14 days vs. 6 days) with longer protocols inducing a slight αCaMKII activation in the NAc. It is also possible that CPP to psychostimulants is accompanied by an increase in αCaMKII phosphorylation in reward-related brain regions other than the NAc [6]. Tan and colleagues, for example, performed CPP to amphetamine at a dose of 2 mg/kg and reported an increase of αCaMKII phosphorylation in the hippocampus [6] as well as an enhancement of hippocampal CaMKII activity [43]. However, using our protocol we could not observe an increase in pαCaMKII in reward-related brain regions such as the VTA and the PFC. 

Social interaction CPP increased αCaMKII activity in the NAc. Consistently, CaMKII in the NAc has been shown to be differently involved in the reinstatement of natural reward-seeking behavior as compared to drug reward-seeking behavior [44]. These results suggest that rewarding social interaction employs the activation of CaMKII in the NAc. In order to specifically identify the NAc subregion involved in the expression of social reward, we infused the rats with the CaMKII inhibitor KN-93 before the test in a concurrent cocaine vs. social interaction CPP. Whereas a KN-93 infusion in the core yielded to equal preference for the cocaine- and social interaction-associated compartments, KN-93 infusion in the shell shifted the preference toward the cocaine-associated compartment. These findings suggest that CaMKII in the shell but not the core subregion of the NAc mediates the expression of social reward. Consistently, when rats were conditioned to cocaine or social interaction, a pre-acquisition bilateral lesion of the NAc shell shifted the preference toward cocaine CPP, thereby suggesting a role of the NAc shell subregion in mediating social interaction reward [18]. In addition, the activation of the NAc shell subregion has been previously shown to be correlated with the time spent in the social interaction-associated compartment during the CPP test [45]. Similarly, the activation of P38 mitogen-activated protein kinase (MAPK) [46] after expression of social CPP and cAMP-response element binding protein (CREB) [47] after reinstatement to cocaine when social reward was made available as an alternative during extinction of cocaine CPP was modulated in the shell but not the core of the NAc. Altogether, these findings highlight the role of the NAc shell in mediating the rewarding effects of social interaction [18]. In line with our study, αCaMKII-T286A mutant female mice showed abnormal social behaviors characterized by decreased social preference and reduced interest in conspecifics of the same sex as compared to their wild type littermates [48]. Moreover, mice with lower total forebrain αCaMKII levels displayed aberrant behavioral phenotypes, including social interaction deficits [49]. Overall, these data indicate that CaMKII affects social interaction.

Although unlikely, we cannot rule out the possibility that the observed shift to the cocaine-associated compartment after KN-93 infusion in the shell of the NAc might be due to enhanced expression of cocaine preference rather than a decrease in social interaction reward. Indeed, evidence suggests a role of CaMKII in psychostimulants’ rewarding and reinforcing effects. Whereas αCaMKII knockout mice show delayed establishment of CPP to cocaine [4] and an attenuated behavioral sensitization to cocaine [14], persistent elevation of autonomous CaMKII in the NAc shell increases both the psychomotor-activating and the rewarding effects of cocaine [10]. This was not observed by Steinkellner and colleagues, who found that the rewarding properties of amphetamine and cocaine were similar in magnitude in αCaMKII-deficient and wild type mice [7]. The latter study suggested that αCaMKII is not required for the rewarding effect of amphetamine and cocaine as measured by CPP. Furthermore, manipulations that inhibit CaMKII activity in the NAc shell have been shown to decrease psychostimulants sensitizing and seeking behaviors [1,8,11,13]. Conversely, manipulations that enhance αCaMKII activity in the NAc shell produce an enhancement in amphetamine-induced locomotion and self-administration [12]. Moreover, it seems that CaMKII activity in reward-associated brain regions is more closely related to the acquisition [3,6,43] rather than the expression of an already-established CPP for psychostimulants [3]. Yet, one study reported a transient impairment of CPP after intra-hippocampal infusion of KN-93 before the CPP test [43]. In addition, intra-VTA CaMKII inhibition before cocaine conditioning, but not before the CPP test blocked cocaine-induced synaptic plasticity in the NAc shell [3]. Thus, CaMKII activity in the VTA regulates cocaine-evoked synaptic plasticity in the NAc shell only during the acquisition of cocaine CPP [3]. Taking into consideration that inhibiting CaMKII in the NAc dampens psychostimulant-associated behaviors, it seems implausible that infusions of KN-93 in the NAc shell would enhance the expression of cocaine preference.

Additionally, we evaluated the time the rats spent in direct physical contact during the last social interaction conditioning session, as “touch” is considered as the most rewarding sensory component in social interaction [42]. As a matter of fact, rats that were assigned to receive vehicle or KN-93 infusion in the NAc shell showed similar time of direct social contact, thereby eliminating the possibility that the shift to the cocaine-associated compartment after KN-93 infusion in the NAc shell subregion was due to an initial decrease in social interaction.

CaMKII is activated by Ca^2+^/CaM through a direct binding mechanism triggering the phosphorylation of critical threonine residues proximal to the CaM-binding site, thereby leading to the auto-activated state of CaMKII. KN-93 is the most broadly used functional inhibitor of CaMKII. It has previously been shown that KN-93 binds directly to Ca^2+^/CaM and not to CaMKII, thereby disrupting the ability of Ca^2+^/CaM to interact with CaMKII and resulting in an effective inhibition of CaMKII activation [50]. It should be noted, however, that some KN-93-based observations might be explained by other Ca^2+^/CaM-dependent CaMKII- independent activities [50,51].

Overall, our results show that social interaction engages CaMKII activation in the NAc shell. We propose that the beneficial effects of social interaction [52] in the NAc shell are dichotomized into rewarding effects mediated via CaMKII and anti-stress effects [19,46,53] mediated via P38 MAPK.

## Data Availability

The data presented in this study should be publicly available and cited in accordance with journal guidelines.
